# Bacterial Control of Pores Induced by the Type III Secretion System: Mind the Gap

**DOI:** 10.3389/fimmu.2016.00084

**Published:** 2016-03-09

**Authors:** Julie Guignot, Guy Tran Van Nhieu

**Affiliations:** ^1^Equipe Communication Intercellulaire et Infections Microbiennes, Centre de Recherche Interdisciplinaire en Biologie (CIRB), Collège de France, Paris, France; ^2^Institut National de la Santé et de la Recherche Médicale U1050, Paris, France; ^3^Centre National de la Recherche Scientifique UMR7241, Paris, France; ^4^MEMOLIFE Laboratory of Excellence and Paris Sciences et Lettres, Paris, France

**Keywords:** SPATE, pore formation, T3SS, membrane repair, cell death

## Abstract

Type III secretion systems (T3SSs) are specialized secretion apparatus involved in the virulence of many Gram-negative pathogens, enabling the injection of bacterial type III effectors into host cells. The T3SS-dependent injection of effectors requires the insertion into host cell membranes of a pore-forming “translocon,” whose effects on cell responses remain ill-defined. As opposed to pore-forming toxins that damage host cell plasma membranes and induce cell survival mechanisms, T3SS-dependent pore formation is transient, being regulated by cell membrane repair mechanisms or bacterial effectors. Here, we review host cell responses to pore formation induced by T3SSs associated with the loss of plasma membrane integrity and regulation of innate immunity. We will particularly focus on recent advances in mechanisms controlling pore formation and the activity of the T3SS linked to type III effectors or bacterial proteases. The implications of the regulation of the T3SS translocon activity during the infectious process will be discussed.

## Introduction

Through secreted proteins, bacterial pathogens have the capacity to induce the formation of pores into eukaryotic host cell membranes. Pore-forming toxins (PFTs) can exert direct cytotoxic effect by irreversibly damaging the plasma membrane, or, at sub-lethal concentrations, can induce cell signaling involved in cytoskeletal reorganization, or in a variety of defense and innate immune responses (1–6). Alternatively, secreted bacterial proteins, such as AB toxins or type III secretion system (T3SS) translocon components, can form transient pores at the plasma membranes to promote the delivery of bacterial virulence factors into the host cytosol. Although host cell responses to various AB toxins have been largely described (7–9), relatively little is known about signaling linked to pore formation mediated by T3SS translocon components.

The T3SS can be viewed as a molecular syringe that upon cell contact, allows the delivery of bacterial effectors directly from the bacterial cytoplasm to the host cytosol [for review, see Ref. (10, 11)]. This system is widely spread among Gram-negative bacterial pathogens and shows conserved structural and functional features. Much of our knowledge has been inferred from extensive studies on the *Shigella*, *Salmonella*, or *Yersinia* T3SSs, and specific characteristics have been reported for other T3SSs, such as those from enteropathogenic (EPEC) and enterohemorrhagic (EHEC) *Escherichia coli*.

As for AB toxins, T3SS-mediated injection of bacterial effectors through eukaryotic cell plasma membranes requires the formation of a “translocation” pore, which occurs upon contact of T3SS with host cell membrane. Cell contact triggers the secretion of translocators proteins through the T3SS: two hydrophobic translocators proteins insert in the host cell membrane to form the so-called translocon, whereas one hydrophilic translocator protein is thought to connect the membrane-inserted translocon and the T3SS needle [for review, see Ref. (12); Table 1]. Here, we will review the responses elicited by host cells, linked to the pore-forming activity of the T3SS, and discuss their role during bacterial pathogenesis.

**Table 1:** **Translocators components in various T3SSs [for review, see Ref. (12)]**.

	Hydrophilic protein	Hydrophobic protein with 2 TM domain	Hydrophobic protein with 1 TM domain
EPEC/EHEC	EspA	EspB	EspD
*Yersinia*	LcrV	YopD	YopB
*Salmonella*	SipD	SipC	SipB
*Shigella*	IpaD	IpaC	IpaB
*Pseudomonas*	PcrV	PopD	PopB

## T3 Translocon and Pore Activity

Upon cell contact, two hydrophobic proteins forming the translocon and containing trans-membrane domains insert into the host cell plasma membrane. Membrane insertion is associated with conformational changes, leading to oligomerization occurring through coiled-coil domain interactions, required for pore formation [Table 1; (12–16)]. Interestingly, coiled-coil domain of translocator proteins share homology with PFT, suggesting common origins and oligomerization mechanisms (17). Although the hydrophilic protein does not integrate in membranes, it is absolutely required for pore activity, possibly by acting as an assembly platform for proper oligomerization of the translocon components (12). The hydrophilic protein is also presumed to provide a molecular link between the translocon and the T3SS needle, through which type III effectors are channeled to get access to the cell cytosol. It is generally admitted that during type III effector translocation into host cells, the translocon is connected to the needle, forming a sealed conduct that does not allow exchange with the extracellular medium. This view is supported by cryo-EM studies showing a continuum between the T3SS and host cell membranes during bacterial infection (18, 19). However, the *Yersinia* type III effector YopH secreted in the extracellular media was shown to translocate into host cells by hijacking translocon components, suggesting that an alternate AB5-like toxin translocation mechanism could also occur for type III effectors (20). Presumably, only translocons detached from T3SS are expected to form pores opened to the extracellular medium. While such considerations remain speculative, and such disconnection may occur following the translocation of injected type III effectors. Studies using artificial membranes have illustrated the pore-forming activity of purified translocon components (21). Although there are numerous evidence demonstrating pore-activity linked to T3SS, structures corresponding to pore-forming translocons are yet to be visualized during bacterial infection (13, 22–25).

Red blood cells (RBCs), which lack internal organelles, are unable to reseal membrane injuries and have been used to demonstrate T3SS-mediated pore formation (26). Release of hemoglobin by RBCs provides a metric for membrane damage linked to pore formation, which, in combination with solute size-dependent osmoprotection experiments, allows to estimate the size of membrane pores. Such experiments indicate that the T3SS induces the formation of pores within host cell membranes with an estimated size ranging from 1.2 to 5 nm, depending on the studies and bacterial systems (27–29). This diameter size is comparable to with that estimated for the inner diameter of the T3SS needle, consistent with a continuum between the needle and the membrane-inserted translocon during the injection of type III effectors. The analysis of the effects of mutations in translocator proteins shows a lack of correlation between T3SS-dependent RBCs’ hemolysis and translocation of type III effectors in epithelial cells (30–34). This suggests that T3SS-dependent pore formation measured by the RBC’s hemolysis assay does not implicate the same requirements as pore formation during translocation of effectors in epithelial cells. These issues are a matter of current debates. Other methods, including the use of fluorescent dyes, have been developed to demonstrate T3SS-dependent pore activity (25, 35).

## Mechanism of T3SS-Dependent Pore Formation

The observations that (i) translocated effectors do not leak into the extracellular medium after injection into cells and (ii) only a minority of cells infected with T3SS-expressing bacteria show dye incorporation assay or K^+^ efflux, point to the inefficient capacity of the T3SS to mediate the formation of pore in nucleated cells (36–38). It was generally thought that as opposed to RBCs, membrane repair in nucleated cells was responsible for this relatively low pore-forming activity. As developed further, it is now clear that bacteria also control pore formation to avoid/or counteract detection by host cells.

In a very recent study, Sheahan and Isberg have identified host cell factors required for *Yersinia* T3SS-associated pore activity. Insertion and assembly of the translocon into the host cell membrane is a more complex process than originally thought, as numerous cytoskeletal and membrane trafficking proteins have been involved (39). This study confirms the key role played by actin and the small Rho GTPase in pore formation (40–42). Unexpectedly, Sheahan and Isberg also identified CCR5, a plasma membrane receptor, as playing a major role in T3-pore formation. CCR5 was recently identified to be a receptor for some PFT, emphasizing the functional homology the between T3 translocon and PFT (43).

## Host Cell Responses to Pore Formation in Plasma Membranes

**Figure 1:**
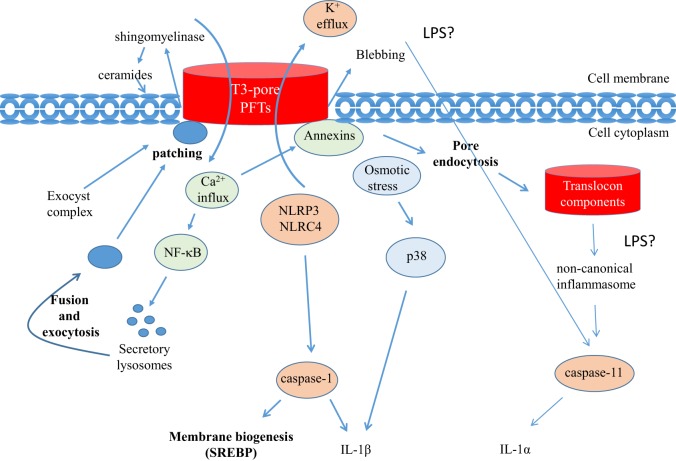
**Membrane repair and inflammasome activation mediated by T3 translocons and PFTs**. Membrane injuries by PFTs or T3 translocon (T3-T) trigger an osmotic stress response, Ca^2+^ influx, and K^+^ efflux that are sensed by host cells. These responses activate innate immune responses and membrane repair mechanisms. K^+^ efflux, or possibly osmotic stress, associated with PFTs leads to the activation of the p38 MAPK and IL-1β secretion. In response to T3-pore formation, inflammasome and caspase-1 activation are also observed in association with K^+^ influx into the translocon component (T3-TC) containing vacuole. Following endocytosis, T3 translocon components can activate caspase-11 through the activation of the non-canonical inflammasome. Membrane repair mechanisms linked to Ca^2+^ influx, lysosomal exocytosis and annexin recruitment are observed. New membrane recruitment to the site of infection by the exocyst complex could also contribute to patch T3-pores.

In response to membrane injuries, cells trigger repair mechanisms involving the detection and removal of damaged plasma membranes. Membrane injuries, such as those induced by PFTs, immediately trigger an osmotic stress response, as well as a Ca^2+^ influx and a K^+^ efflux that are sensed by host cells (4, 44–46). These responses activate MAP kinase signaling, inflammasomes, and NF-κB activation, which in turn lead to the elicitation of inflammatory and innate immune responses (Figure 1). Such signaling also activates membrane repair mechanisms: K^+^ efflux triggers NLRP3 activation, leading to the recruitment of Caspase-1 (IL-1-converting enzyme) (47). Caspase-1 has a dual effect; it cleaves pro-IL-1β to generate mature IL-1β and stimulates the sterol regulatory element-binding proteins (SREBPs) to promote membrane biogenesis (48). Fast-acting cortical membrane repair involving exocytic and endocytic processes are also well described (49, 50). Ca^2+^ influx triggered by pore formation is sensed by synaptotagmin, a Ca^2+^ sensor present at the surface of lysosomes. Intracellular Ca^2+^ increase determines the synaptotagmin-dependent fusion of specialized lysosomes, named secretory lysosomes, in large vesicles. These vesicles fuse with wounded membranes, a process that contributes to the patching of pores at the plasma membranes (26, 49, 50). Fusion of secretory lysosomes with wounded plasma membranes also leads to the release of lysosomal enzymes, such as sphingomyelinases, into the medium. Sphingomyelinases hydrolyze sphingomyelin to form ceramides that induce membrane curvature. This curvature is thought to initiate endocytosis of damage membranes that are subsequently targeted to intracellular degradation. Endocytosis has been proposed as an active repair mechanism of membrane damaged by PFTs (44). Ca^2+^ influx also leads to the binding of cytoplasmic annexins to the plasma membrane, resulting in the connection of the membrane to actin network. Annexin A5 was also shown to form a network limiting diffusion at the site of membrane injury (51). Ca^2+^ influx has also been associated with the annexin-dependent blebbing of the plasma membrane leading to the shedding of vesicles containing pores mediated by PFT in the extracellular milieu (52–54).

## Characterizing Signals Linked to Membrane Insertion and Pore Activity of the T3 Translocon

Identifying signals that specifically associated with the T3SS translocon is challenging because it is also required for the translocation of type III effectors, many of which being reported to regulate innate immune responses. Furthermore, various microbial structures, including structural components of the T3SA, act as pathogen-associated molecular patterns (PAMPs) and are sensed by host cells to induce innate immune responses that are not directly associated with translocon insertion into host membranes (55, 56). To identify translocon-specific signals, studies have reported the use of bacterial mutants lacking all type III effectors (effectorless strain) and/or using cells lacking the two main TLR adaptor proteins (MyD88^−/−^ and Trif^−/−^) and hence, deficient for TLR signaling downstream of PAMPs. Such studies showed that the insertion of translocon components into the host plasma membrane activates an innate immune response that differs depending on the cell type (42). Insertion of the *Yersinia* translocon is associated with NLRP3 and NLRC4 activation with downstream signaling events leading to caspase-1 activation and IL-1β production (57, 58). The T3SS-dependent activation of NLRC4 has also been observed for *Shigella*, *Salmonella*, and *Pseudomonas* (59, 60). For *Salmonella and Pseudomonas*, such NLRC4 activation was shown to depend on T3SS-dependent pore formation and K^+^ efflux (37). Activation of the non-canonical caspase-11 (caspase 4 in humans) inflammasome has also been described to be dependent on the T3SS, although recent evidences indicate that bacterial LPS could account for caspase-11 activation (61–64).

The cytosolic presence of translocators, rather than pore formation, has also been described to activate the inflammasome (65). The detection of translocator components in the cytosol has been attributed to the cytoplasmic tail of one of translocators following its insertion in the plasma membrane, or, alternatively, to the endocytosis of the pore-forming translocon complex. In both cases, cytosolic access of T3 translocon components leads to canonical NLRP3 and non-canonical caspase-11 activation, similar to what has been described for cytosolic PAMPs (62, 65). Consistent with a role for translocon endocytosis, Senerovic et al. have described that the purified translocator component IpaB oligomerizes in membrane and forms ion channels promoting K^+^ influx upon internalization within endosomes, responsible for macrophages cell death. In this case, translocon-dependent K^+^ influx into vacuoles may affect endolysosomal membranes’ integrity, leading to caspase-1 activation downstream of the NLRC4 inflammasome (66).

Perhaps most indicative of T3SS-dependent pore-forming activity, membrane repair mechanisms are also activated upon bacterial infection. In response to Ca^2+^ influx linked to T3SS-dependent pores, synaptotagmin-dependent lysosomal exocytosis has been reported in *Salmonella* and *Yersinia* infected cells (39, 67). During infection, *Salmonella* and EPEC also trigger the recruitment and activation of the Ca*^*2*^*^+^-sensors annexins at the site of bacterial attachment (68–73).

## Bacterial Mechanisms of Avoiding Cell Death Linked to T3SS-Mediated Pore Formation

Invasive bacteria, such as *Salmonella* or *Shigella*, promote their uptake in vacuole, resulting in a process leading to the removal of membrane-inserted translocons from the plasma membrane. This “self-removal” of membrane-inserted translocons may represent an additional factor contributing to the difficulty in detecting pore formation in epithelial cells infected by these bacteria. To minimize plasma membrane damages linked to T3 translocons, bacteria that multiply extracellularly have developed multiple strategies against inflammatory cell death. Injected type III effectors may downregulate cell death and inflammatory signals, by interfering with initiator or effector caspases and NLRC4 inflammasome activation (74). The role of these type III effectors has been recently reviewed elsewhere (57, 58, 75–77). Here, we will mostly discuss the bacterial regulation of T3SS-dependent pore formation.

**Figure 2:**
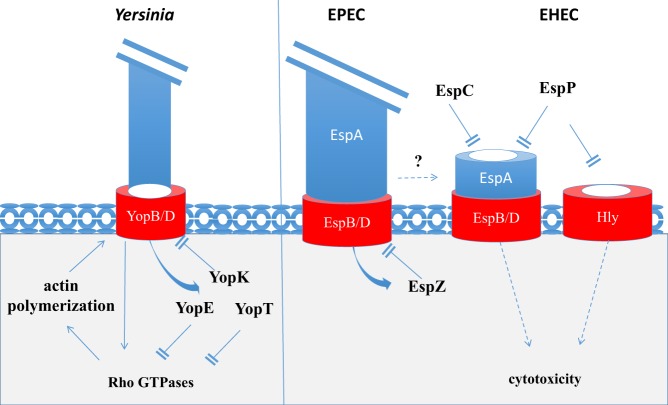
**Bacterial effectors regulating T3-pore formation**. Upon cell contact and T3SS activation, the *Yersinia* YopB/D translocon components activate Rho GTPases leading to the polymerization of actin and T3-pore formation. The injected T3 effectors, such as YopK, YopE, and YopT, downregulate T3-pore formation and effector translocation. YopK directly acts on the T3 translocon. YopE and YopT inhibit RhoGTPases. EspZ shares an activity related to that of YopK by binding to the EPEC T3 translocon, inhibiting T3-pore formation and effector injection. EspC downregulates T3 pore by degrading the translocator components EspA/D, an activity shared by the EHEC EspP. EspP also downregulate the Hly PFT inserted in plasma membranes.

In *Yersinia*, at least three different type III effectors, such as YopK, YopE, and YopT, regulate T3SS-dependent pore formation and effector injection into host cells. The translocon component YopB activates both pro-inflammatory response and the small Rho GTPase, Ras (42, 78). YopB/D translocon insertion, in cooperation with invasin-beta1 integrin signaling, activates multiple Rho GTPases leading to actin polymerization, a step absolutely required for the *Yersinia* T3SS-dependent pore formation in the plasma membrane. The role of actin polymerization in the formation of the *Yersinia* T3SS-dependent pore is not clear but might reflect the importance to affix plasma membrane while translocon is inserted, or the translocon disconnection from the T3SA following effector injection in host cells. Among injected effectors, YopE and YopT display pore inhibition activity through the downregulation of several Rho GTPases (RhoA, RhoG, Rac1, and CDC42), linked to a GAP and protease activity toward these GTPases, respectively. Inhibition of Rho GTPase activity associated with actin depolymerization not only prevents pore formation but also reduces effector translocation. YopK also negatively regulates injection of type III effectors and cytotoxicity. As opposed to YopE and YopT regulating the T3SS activity through their action of Rho GTPases, YopK binds to the translocon and may directly clot it or induce conformational changes leading to translocation blockage (76). Although sharing little primary sequence homology with YopK, the EPEC/EHEC type III effector EspZ displays a similar activity (Figure 2). EspZ was shown to interact with the EPEC-translocon component EspD and prevents cell death by preventing the translocation of T3SS effectors into infected cells (79).

## Proteolytic Degradation of T3-Pores by a Bacterial Serine Protease Autotransporter of Enterobacteriaceae

More recently, our group has reported a novel mechanism controlling T3SS-mediated pore formation and cytotoxicity induced by EPEC and EHEC (38). In addition to the T3SS, EPEC secretes other bacterial toxins involved in virulence. Among these, EspC, a protease belonging to the serine protease autotransporter of enterobacteriaceae (SPATE) family (80, 81), was shown to degrade the T3SS translocon components following contact with epithelial cells, thus downregulating T3SS-dependent pore formation and cytotoxicity. In EPEC, the hydrophilic translocator component EspA polymerizes into a filament connecting the T3SS needle to the translocon that is composed of the EspB and EspD hydrophobic proteins. EspC appears to preferentially target EspA associated with EspD. Since EspC does not prevent type III effector injection, it may recognize a specific conformation of EspA/D corresponding to a T3SS “by-product” with potential cytotoxic activity. Interestingly, EspP, the EspC hortologue in EHEC has been involved in the proteolytic degradation of the *E. coli* hemolysin Hly, a pore-forming cytolysin (82). The cleavage of Hly by EspP occurs in the region of the hydrophobic domain and lead to the inactivation of its pore-forming activity.

## Epithelial Cell Death Linked to T3SS-Pores

Depending on the cell type and the extent of pore formation, membrane lesions can lead to apoptotic or necrotic cell death. It has been suggested that pores detected in epithelial cells infected with effectorless *Yersinia* or an EPEC *espC* mutant result from unsealed translocons similar to those found in membranes of erythrocytes. With the exception of T3SS-dependent cell death induced by *Yersinia*, which appears to implicate distinct pathways, T3SS-dependent cytotoxicity appears to be caspase independent (38, 79, 83, 84). Epithelial cells dying from T3SS-dependent unregulated pore formation show nuclear shrinkage without signs of nuclear fragmentation, consistent with non-apoptotic cell death (38, 79, 83, 84). The precise mechanism implicated in this T3SS-dependent death is unknown. In unrelated studies, however, nuclear shrinkage and caspase-independent cell death have been linked to the activation of phospholipase A2 (PLA2) (85). Interestingly, PLA2 activation associated with K^+^ efflux and/or Ca^2+^ influx triggers IL-1 β secretion (86, 87), as observed for T3SS-dependent pore formation. Nuclear shrinkage may correspond to a common response to membrane insults induced by PFTs and T3SS-dependent unregulated pore formation (88, 89).

## Concluding Remarks and Perspectives

As reviewed here, T3SS-expressing bacteria have developed a diversity of mechanisms to downregulate the formation of pores linked to the activity of T3SS translocon, reflecting the importance of this process in the pathophysiology of bacterial infections. In the absence of such translocon regulatory processes, a variety of inflammatory and death processes can be induced, depending on the bacterial pathogen. Although the insertion of T3SS-translocons during type III effector injection may induce a common canonical response associated with the activation of the NLRC4 inflammasome and eventually, necrotic cell death, these responses may be subsequently further tuned by other bacterial effectors. Deciphering how these signals integrate during the course of the bacterial infectious process represents a challenge needed to be addressed in future studies. Understanding how the T3SS pore formation and injection of effector is regulated could also lead to the development of innovative therapeutic molecules, widening the spectrum of currently studied T3SS inhibitor (90, 91).

## Author Contributions

JG wrote the manuscript, and GN edited the manuscript.

## Conflict of Interest Statement

The authors declare that the research was conducted in the absence of any commercial or financial relationships that could be construed as a potential conflict of interest.
